# Formation of Monolithic Ion-Selective Transport Media Based on “Click” Cross-Linked Hyperbranched Polyglycerol

**DOI:** 10.3389/fchem.2019.00484

**Published:** 2019-07-10

**Authors:** Tobias Abrahamsson, David J. Poxson, Erik O. Gabrielsson, Mats Sandberg, Daniel T. Simon, Magnus Berggren

**Affiliations:** ^1^Laboratory of Organic Electronics, Department of Science and Technology, Linköping University, Norrköping, Sweden; ^2^RISE Acreo AB, Norrköping, Sweden

**Keywords:** hyperbranched polyglycerol, polyelectrolyte, multi-functionalization, thiol-ene, cross-linking, ion-selective, electrophoretic transport

## Abstract

In the emerging field of organic bioelectronics, conducting polymers and ion-selective membranes are combined to form resistors, diodes, transistors, and circuits that transport and process both electronic and ionic signals. Such bioelectronics concepts have been explored in delivery devices that translate electronic addressing signals into the transport and dispensing of small charged biomolecules at high specificity and spatiotemporal resolution. Manufacturing such “iontronic” devices generally involves classical thin film processing of polyelectrolyte layers and insulators followed by application of electrolytes. This approach makes miniaturization and integration difficult, simply because the ion selective polyelectrolytes swell after completing the manufacturing. To advance such bioelectronics/iontronics and to enable applications where relatively larger molecules can be delivered, it is important to develop a versatile material system in which the charge/size selectivity can be easily tailormade at the same time enabling easy manufacturing of complex and miniaturized structures. Here, we report a one-pot synthesis approach with minimal amount of organic solvent to achieve cationic hyperbranched polyglycerol films for iontronics applications. The hyperbranched structure allows for tunable pre multi-functionalization, which combines available unsaturated groups used in crosslinking along with ionic groups for electrolytic properties, to achieve a one-step process when applied in devices for monolithic membrane gel formation with selective electrophoretic transport of molecules.

## Introduction

Organic bioelectronics (Wei, 1998; Berggren and Richter-Dahlfors, 2007; Someya et al., 2016) comprises the research and engineering field in which the coupling of biomolecules and electronic charges, in organic electro-active materials, is utilized to achieve sensing or actuation of processes and reactions in various biological systems. During the last decade, major interest in this field has included the development and engineering of devices that convert electronic addressing signals into the delivery of small-sized charged biomolecules. In one embodiment, planar drug delivery electrodes, based on conjugated polymers pre-loaded with a biomolecule, have been explored (Kontturi et al., 1998; Abidian et al., 2006; Wadhwa et al., 2006). Upon electronically addressing these electrodes, their oxidation state changes, triggering release of the pre-loaded biomolecule. This effect is driven by the changes in electrostatic and morphological properties of the electrode bulk upon switching. In another embodiment, the mixed ion-electron conduction of two separated conjugated polymer electrodes are combined with the selective ion transport of a membrane to form a class of electrophoretic drug delivery devices, called organic electronic ion pumps (OEIPs) (Isaksson et al., 2007; Simon et al., 2009; Arbring Sjöström et al., 2018). By addressing the two polarizable electrodes of the OEIP an electric field is established along the ion selective channel, enabling the selective delivery of small (≲200 g/mol) charged biomolecules, transported from a reservoir to a target system. Such OEIP devices have successfully been explored to deliver neurotransmitters and ions in various applications, especially targeting actuation and suppression of neuronal processes and signaling *in vitro* (Tybrandt et al., 2009; Williamson et al., 2015; Jonsson et al., 2016) and *in vivo* (Simon et al., 2009; Jonsson et al., 2015; Proctor et al., 2018).

The monolithic ion-selective channel of an OEIP should exhibit a high and selective conductivity for the desired charged (bio)molecule, along with manufacturability of miniaturized structures to enable application *in vitro* or *in vivo*. Furthermore, to match the scale of target biological systems and provide the highest degree of proximity and spatiotemporal precision, OEIPs are generally fabricated with ion-selective channels on the micrometer scale (width, approaching the scale of a single cell) and thickness of hundreds of nanometers (based on their thin-film nature). To date, most OEIPs and related iontronic devices (Arbring Sjöström et al., 2018) have utilized linear polyelectrolyte systems, such as polystyrenesulfonate or quaternized poly(vinylbenzyl chloride), processed into features using classical thin film processing techniques. As produced, these features are dry and when immersed into electrolytes and media, the ion-selective conductor swells due to considerable uptake of water and electrolyte solutes, leading to frequent mechanical failure of the devices and low production yield. Recently, our group has demonstrated capillary-based OEIPs, where the monolithic ion transport channel is formed inside a free-standing glass capillary (Poxson et al., 2019; Seitanidou et al., 2019). These devices take advantage of the glass capillary (generally ≤ 60 μm outer diameter) as both the encapsulation and substrate for the ion channel. As such, the encapsulation is in place during fabrication, enabling the channel to remain hydrated (if stored properly) continuously from fabrication to end-use.

Formation and operation of ion-selective monolithic ion transport media in microfluidic or capillary-based channels places a range of demands on the design of the molecules and reactions forming the given transport medium. Most importantly, the medium should comprise a continuous porous structure through the monolithic body where the pore size should permit mobility of the targeted charged molecules. The pores should, however, not be so large as to permit macroscopic flow. Further, the number density of fixed charges should be sufficient for ion-selective transport, and the distribution of charges should be as homogeneous as possible. Moreover, the monolithic ion transport medium should be obtained by polymerization of molecules via reactions that can be triggered by external stimuli such as heat or actinic irradiation under atmospheric conditions to form bonds that are stable at the extreme pH-values that may occur in ion transport devices (Seitanidou et al., 2017). For straightforward filling of the microfluidic channel, the composition forming the ion transport medium (monomers and polymerization initiators) should exist as a single-phase fluid to enable filling using a minimum of solvent. Mechanically, the formed monolithic ion transport medium should exhibit a cohesive strength sufficient to tolerate solvent exchange and exhibit a good adhesion to the channel walls. We have selected thiol-ene click coupling to form the cross-links between monomers upon irradiation, since the thiol-ene coupling reaction tolerates the presence of air oxygen and proceeds to produce cross-links that are stable at the pH-values reached during OEIP operation (Hoyle and Bowman, 2010). As a structural building block, we have selected polyfunctional hyperbranched and dendrimeric molecules. These polyfunctional dendrimers can be substituted with groups for cross-linking as well as ionic groups with controlled degrees of substitution. It is of course important that the structural unit, the dendrimer, is formed by bonds that are stable to conditions of an OEIP under operation. Therefore, we have opted for polyether dendrimers having hydroxyl terminal groups since the ether groups are relatively stable to extreme pH values.

The use of thiol-ene chemistry calls for the presence of carbon-carbon double bonds as well as thiol groups. We have selected to functionalize the dendrimers with allyl groups for coupling with a second molecule, a thiol-terminated polyether. In addition to allyl-groups, the dendrimers are functionalized with groups carrying either positive or negative charges. To form the covalently cross-linked ion-transport medium with thiol-ene coupling, radical initiators must also be present, in addition to the allyl-functional dendrimers and thiol-functionalized cross-linkers, and these components must exist as a single-phase mixture with blending at the molecular level in the composition. For this reason, there is a need to add a minimal amount of solvent to form the single-phase composition. The amount of solvent should be minimal and constitute a minority component in the composition to avoid formation of large voids in the formed ion-transport channel, which would lead to breakdown of Donnan exclusion (charge selectivity) (Kontturi et al., 2008).

Here we report a new type of material synthesized and characterized based on a hyperbranched polyglycerol foundational structure, which has been multi-functionalized with different degrees of allyl and triphenylphosphonium groups. The click chemistry polymerization between the cationic polyelectrolyte mixed with tri-functional polythiol cross-linker has been achieved via thermal and photo-initiation. The sol-gel process of the macromolecules has formed insoluble free-standing membranes, capable of selective electrophoretic transportation of large aromatic molecules in OEIP structures. Due to solubility properties of the hyperbranched polyelectrolyte, these types of gel membranes can be prepared with a minimal amount of organic solvent with pre-hydrated states for OEIPs, iontronics, and other such organic (bio)electronic devices.

## Results

### Synthesis and Structural Characterization

**Scheme 1:**
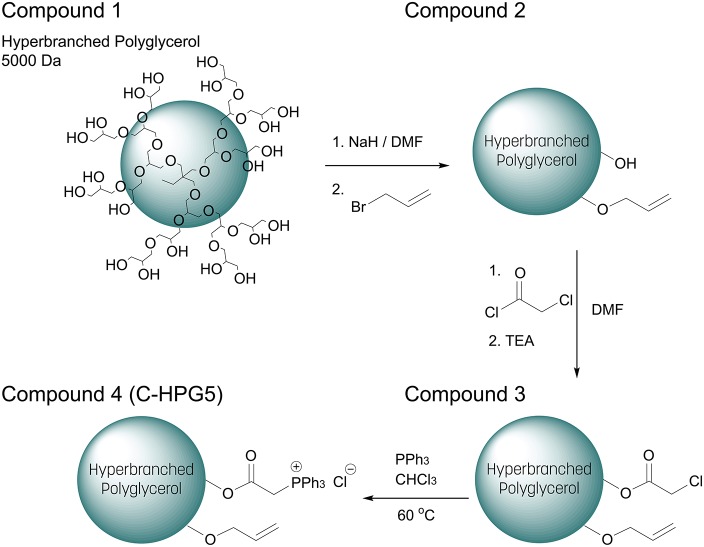
Synthetic multi-functionalization of hydroxyl end-groups of (1) Hyperbranched Polyglycerol 5000 Da (simplified structural representation) with (2) allyl and (3) chloroacetate. (4) Chloride substituted with triphenylphosphine into charged phosphonium (C-HPG5).

The multi-functionalization synthesis of the hyperbranched polyglycerol (5,000 Da) was carried out step wise, utilizing the multiple terminal hydroxyl groups of the polymer (Scheme 1). In the initial step, allylation was performed on the polymer resulting in a degree of substitution of ~ 10% (Figure S1a) and distinct alkene carbon signals were detected in C-NMR (Figure S1b). This characterization was calculated via ^1^H-NMR spectroscopy where the integral ratio of the specific functionalities' protons was compared to the integral ratio of the core polymer's structure protons (Equation 1), assuming the correlation with the number of terminal groups equal to the number of repeating units of the polymer (Gode et al., 2006; Burakowska and Haag, 2009).

(1) $$\begin{array}{l} DS\left(\%\right)=\left(\frac{I_{H-func.}}{H_{func.}}/\frac{I_{H-core}}{H_{core}}\right)\times 100 \end{array}$$

The remaining hydroxyl groups were then converted into esters by introducing chloroacetyl functionality, though signal overlapping occurred between the newly introduced protons located at the α-chloroacetate and the previously incorporated methylene protons neighboring to the alkene groups, thus somewhat obstructing the values of integration. Regardless, a well-defined characteristic peak was observed (Figure S2a) with an integral magnitude in accordance with a high degree of functionalization of ~90% chloroacetate (Figure S2a equation). Likewise, the functionalization shifts of the outer layer protons located at secondary and tertiary carbons which previously had hydroxyl (primary, secondary) functionality, became ester (primary, secondary) functionalities resulting in downfield shifts. This made the further estimation of the different functionalities much more difficult. The hyperbranched polyglycerol's solubility changed drastically with the incorporation of the hydrophobic α-chloroacetylate groups to the polymer, rendering it insoluble in hydrophilic solvents, emphasizing the crucial role of the surface functionalities of the hyperbranched polymers role in interacting with its surrounding environment (Türk et al., 2007; Thota et al., 2016).

**Figure 1:**
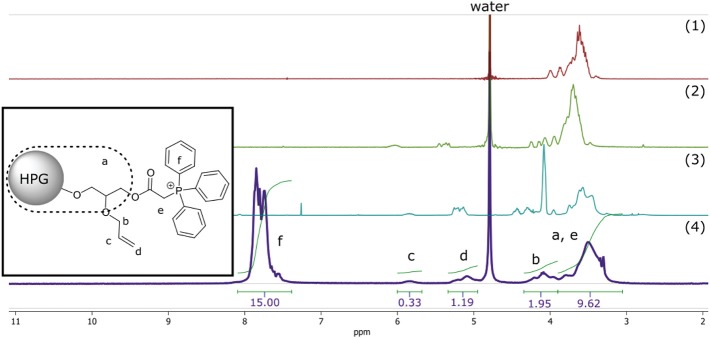
^1^H-NMR of compound 1–4. Simplified representation of C-HPG5 chemical structure with assigned and integrated peaks (4). Assigned protons of allyl (b and d) integral signals regions overlap with outer core protons of HPG (a) depending on functionality.

The relationship between surface functionalization and solubility was also observed in the final synthetic step of the polymer where ionic functionality was introduced to the macromolecular structure—rendering it again soluble in polar solvents—by nucleophilic substitution of the α-chloride with triphenylphosphine, quaternizing the phosphor into positively charged phosphonium (Matveeva et al., 2003). This final synthetic step resulted in cationically-functionalized hyperbranched polyglycerol with molecular weights of 5 kDa (C-HPG5, Scheme 1, Compound 4). The values obtained from the NMR peak integration of the introduced triphenyl functionality (Figure 1) lead to yields of the quaternization reactions between 60 and 70% (Figure S3), though solvent peak signals in the polymer region along with functionality shift overlap complicate this estimation.

Peak splitting was observed in the NMR spectra for the peak associated with the six-membered phenyl rings of the phosphonium, which can be related to the presence of different structural conformation over phosphor (Aganova et al., 2014) seen in ^31^P- and ^13^C-NMR. ^31^P-NMR studies of C-HPG5 also showed solvent-dependent peak splitting (Figure S3c). There was a partial split of the phosphonium signal seen in methanol-d_6_, but not exhibited in D_2_O.

The amphiphilic nature displayed of the triphenylphosphonium hyperbranched polyelectrolyte (C-HPG5) made it easily solubilized in very small amounts of methanol solution with up to 3 g/mL (80 wt %) of C-HPG5 polymer. This demonstrates high solubility capabilities of the hyperbranched polyelectrolyte with significant amounts of charged surface functionalities (Kłos and Sommer, 2010). In combination with the hydrophobic triphenyl groups, this renders the C-HPG5 polymer soluble/processable from both protic and aprotic solvent mixtures. This important feature was utilized in the subsequent cross-linking, where components such as initiators and cross-linking agents exhibit much more lipophilic solubilities. The versatile solubility also makes the C-HPG5 polyelectrolyte able to interface the system of hydrophilic cationic charges and hydrophobic interfaces in low amounts of solution.

### Thermal Cross-Linking

**Figure 2:**
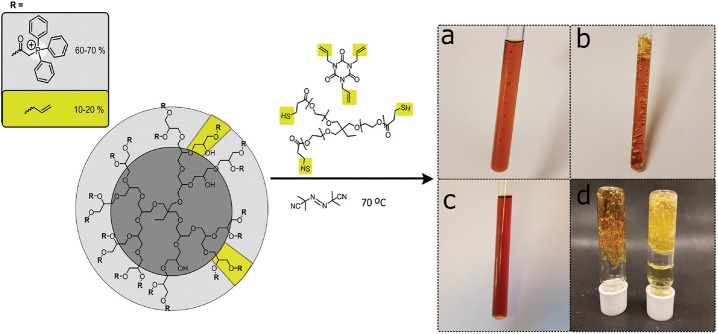
High concentration liquid mixtures of C-HPG5 polymer, Thiocure^®^ ETTMP 1300, TATATO, and AIBN for thermal-initiated cross-linking and formation of gel membranes. Highlighted cross-linking units in yellow. **(a)** Initial heating stage with small bubbles formed due to boiling methanol. **(b)** Finalized cross-linking in methanol resulted in a porous insoluble gel network, while **(c)** in DMSO a more consistent gel was formed due to the higher boiling point solvent. **(d)** The cross-linked gels were able to withstand solvent swelling in methanol (left) and solvent exchange to H_2_O (right).

To obtain the cross-linked hyperbranched polyglycerol electrolyte, the triphenylphosphonium and allyl functionalized HPG (C-HPG5, Compound 4) was first mixed with Thiocure ETTMP 1300 (ethoxylated-trimethylpropan tri(3-mercaptopropionate)) with AIBN (2,2′-azobis(2-methylpropionitrile)), and TATATO (1,3,5-triallyl-1,3,5-triazine-2,4,6(1H,3H,5H)-trione) in water/methanol mixtures or DMSO, applying high concentration kinetics before thermal-initiated crosslinking (Figure 2).

The thiol-ene click chemistry reaction (Hoyle and Bowman, 2010; Trey et al., 2010) allowed the formation of large amounts of the cross-linked insoluble free-standing gel from versatile, inexpensive starting materials. Our initial experiments showed that synthetic mixtures under high concentration kinetic control successfully cross-linked with both anionic or cationic functional groups (e.g., sulfonate or phosphonium) by applying photo-initiated surface chemistry for the formation of anionic or cationic exchange membrane (AEM or CEM) gel materials. Unfortunately, we found that the thermally-initiated cross-linking reaction did not proceed in solution for the cationic C-HPG5 without addition of TATATO (Kasprzak et al., 2009), independent of the inclusion or exclusion of the thiol cross-linker Thiocure 1300 (Table 1, TM1-3). With several different reaction conditions applied, the concentration was observed to play a crucial role for the success of the thermally-initiated sol-gel: lower concentration of C-HPG5 polyelectrolyte combined with the same equivalents of reactants (as TM1) did not lead to successful gel formation (Table 1, TM4-5). To ensure that the final gels consisted primarily of the C-HPG5 polyelectrolyte material, the concentrations of the other components were reduced to levels corresponding to theoretically calculated 1:1:1 ratios of allyl-thiol-allyl for the three components, in accordance with the ~10–20% allyl functionalized C-HPG5. This formulation did successfully cross-link, emphasizing the high concentration kinetics requirement (Table 1, TM6, Figure 2b). The exact concentration limit of the polyelectrolyte for the reaction wasn't defined in methanol, though attempts with similar reaction conditions in DMSO proved able to form a continuous gel at 1.13 g mL^−1^, nearly half the concentration as T1-3 or T6-7 (Table 1, TD1-2, Figure 2c). The lower degree of porosity was attributed to the higher boiling point of the solvent, which was also observed in TM1 with the crosslinking conducted in either methanol or 1-propanol (Figure S4).

**Table 1:** Thermal cross-linking formulations.

**Thermal cross-link**	**C-HPG5 (g mL^**−1**^)**	**Thiocure 1300 (g mL^**−1**^)**	**TATATO (g mL^**−1**^)**	**AIBN (g mL^**−1**^)**	**Gel formation**
TM1_MeOH_	2	2	0.11	0.34	Yes
_w/10%H20_	2	2	0.11	0.34	Yes
_w/propanol_	2	2	0.11	0.34	Yes
TM2_MeOH_	2	-	0.11	0.34	No
TM3_MeOH_	2	2	-	0.34	No
TM4_MeOH_	0.2	0.2	0.01	0.03	No
TM5_MeOH_	0.5	0.13	0.03	0.07	No
TM6_MeOD−d4_	2	0.56	0.10	0.10	Yes
TM7_MeOH, cata._	2	2	0.11	0.01	Yes
TD1_DMSO_	1.13	1.13	0.06	0.19	Yes
TD2_DMSO−d6, *NMR*_	1.13	0.29	0.06	0.06	Yes

**Figure 3:**
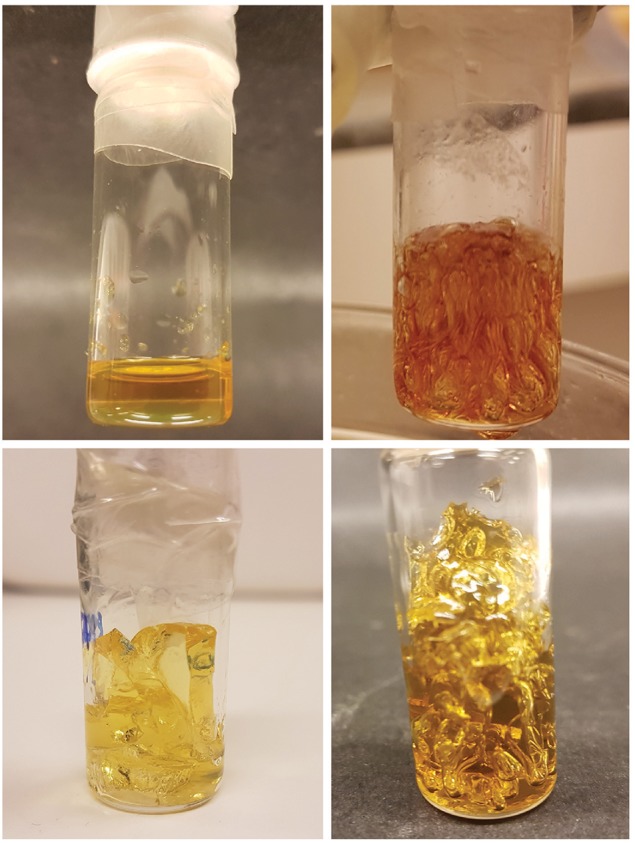
Top row: Catalytic amount of AIBN (0.2 wt%) resulted in a more continuous gel (left) while high amounts (6.5 wt%) led to more porosity (right). Bottom row: Respective gels swelled in methanol. The catalytic gel (left) was cut into pieces to illustrate that an insoluble gel formation had occurred.

Experiments with catalytic amounts of AIBN (0.2 wt%) were also conducted (Table 1, TM7). This formulation resulted in an insoluble gel, though without high amounts of visible porosity compared to the TM1 (6.5 wt%, Figure 3) formulation. Gelling with high amounts of AIBN initiator exhibited pronounced pores, formed from the boiling of solvent during the solidification. However, reducing AIBN concentration to catalytic amounts enabled incorporating methanol more continuously into the gel. N_2_-gas evolution from AIBN initiator breakdown may also play a role in pore formation.

### NMR Monitoring of Thermal Cross-Linking

**Figure 4:**
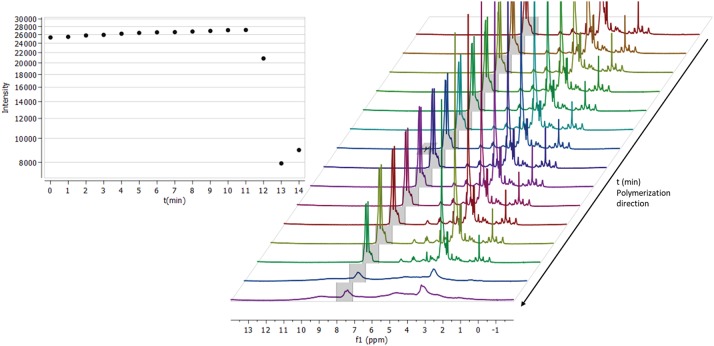
Thermal initiated crosslinking with C-HPG5 at 70°C in DMSO, monitored by collecting 1H-NMR (300 MHz) spectra every minute (right). Integral of the triphenyl functionalities intensity (gray region in spectra) plotted against time (left). When the reaction is initiated it occurs at an extremely fast rate, distinguished by the rapid loss of the signal intensity.

Components were combined in DMSO-d_6_ according to formulation TD2 (Table 1) and transferred into an NMR-tube. Following 5 min of sonification and deaerating with N_2_-gas, the tube was sealed and placed inside a Varian Oxford 300 MHz NMR spectrometer. When the tube had reached a temperature of 70°C, the first spectrum was collected, and we proceeded to monitor the crosslinking reaction. The process was carried out by locking all the parameters according to the initial spectrum (to prevent re-shimming) and spectra were then collected every minute (Figure 4). After the heating had proceeded for 11 min with no significant change in intensity between spectra, the gelification process began. The sol-gel reaction proceeded with a rapid velocity and with the spectrum collected at minute 12, a change in the integral intensity was already observed (intensity ~27,000 → 20,000). Within the time frame of 2 min, the entire solution had solidified, characterized by a substantial loss of signal intensity (Figure 4, 12–14 min) and broadening of the spectrum's overall signatures. Due to a rapid build-up of high pressure inside the tube during the gelification process, the monitoring was terminated after 14 min. A somewhat increased integral intensity (~8,000 → 9,000) between the last two time points (13–14 min) could be seen, which is related to the overall broadening of signal intensity across the entire spectrum.

### Photo-Initiated Cross-Linking

A series of free-standing transparent membranes were synthesized by drop-casting solutions (Table 2) on glass, Teflon, or gold substrates followed by UV exposure for 10 min at 365 nm (10 mJ/cm^2^). These membranes were then peeled off from the substrate surfaces, displaying soft, flexible properties while remaining intact after soaking in solvents such as deionized water and methanol along with solvent exchange (Figure S5). It was also observed that when the membranes had been equilibrated in deionized water, the mechanical strength was somewhat reduced, resulting in more brittle—but still flexible—gels.

**Table 2:** Photo-initiated cross-linking formulations.

**Photo cross-link**	**C-HPG5 (g mL^**−1**^)**	**Thiocure 1300 (g mL^**−1**^)**	**TATATO (g mL^**−1**^)**	**Irgacure 2959 (g mL^**−1**^)**	**Gel formation**
UVC1_MeOH_	1	1	0.06	0.28	Yes
UVC2_MeOH_	0.5	0.5	0.03	0.12	Yes
UVC3_MeOH/H20_	0.5	0.26	0.03	0.007	Yes
UVC4_MeOH/H20_	0.5	0.13	0.03	0.03	Yes

In the series of drop-casting formulations (Table 2), the Thiocure 1300 component was reduced so as to reach the theoretically calculated 1:1:1 ratio with the unsaturated allyl functionality (~10 mol %) of the C-HPG5 in relation to the allyl of TATATO and the thiol. We thus aimed to achieve a membrane consisting primarily of C-HPG5 polymer (UVC4). We also reduced the composition concentration (0.5 g/mL C-HPG5) to investigate if the photo-initiated surface crosslinking could proceed at lower concentrations in comparison with thermally-initiated cross-linking (UVC2-4). These formulations did proceed to gelification, and in the two latter cases we were able to use a 50/50 blend of water/methanol as solvent (Figures S5, S6). The addition of water was intended to both reduce the factor of organic solvent and to ensure less solvent evaporation (higher boiling point) during the UV-curing process. Not only was membrane formation still possible with the 50/50 water/methanol solvent, but the resulting membranes were in a pre-hydrated state due to incorporation of water. With minimum amounts of other reagent components such as catalytic amounts of photo-initiator (UVC3) and a predominant C-HPG5 composition mixture (UVC4), we demonstrate the ability to formulate and crosslink into membranes at both low and high concentrations. This is due to the solubility of the cationic hyperbranched polyelectrolyte, whereas the limiting factor otherwise lies with the miscibility of the initiator and cross-linker.

### Utilization in Organic Electronic Ion Pumps

**Figure 5:**
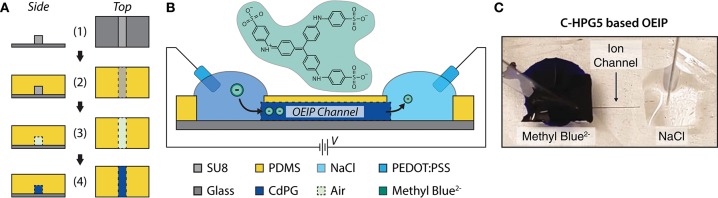
C-HPG5-based OEIP device. **(A)** Schematic of OEIP MIMIC fabrication: (1) SU-8 is patterned on glass substrate; (2) PDMS cured over SU-8 pattern; (3) PDMS with molded void is transferred to a UV-ozone-treated glass substrate; (4) C-HPG5 is filled into PDMS void via capillary force and UV cross-linked. **(B)** Schematic profile of fabricated C-HPG5 based OEIP and chemical structure of methyl blue. **(C)** Photograph of C-HPG5-based OEIP during operation. Methyl blue in the source reservoir is electrophoretically transported through the C-HPG5 AEM channel toward the target electrolyte, NaCl(aq), due to applied voltage between the PEDOT:PSS electrodes. At the time point of the photograph, the front of methyl blue had nearly reached the target electrolyte.

To characterize the C-HPG5 membrane's anion transport capabilities, we fabricated OEIPs using a micro-molding in capillaries (MIMIC) technique (Kim et al., 1996) (Figure 5A). OEIPs were manufactured by pouring and curing PDMS into ~2 mm thick films on a glass master substrate with a (photolithographically) pre-patterned SU-8 pattern on top of it, defining the ion channel geometry (Figure 5A-1). The width and height of the SU-8 pattern were measured to be 85 and 43 μm, respectively, using an optical profilometer (Figure S7). The PDMS was cut into small blocks, consisting of two separated electrolyte reservoirs connected by a capillary channel with an average length of 7.1 mm. After surface activation by treatment with O_2_ plasma, the PDMS was bonded to glass substrates (Figure 5A-3). The channels were filled with C-HPG5 cross-linking solution (formulation UVC4) by capillary force according to the MIMIC technique (Figure S8a). Following UV cross-linking as described above, the C-HPG5-based AEM was formed (Figure 5A-4) where the homogeneity and continuity was studied using scanning electron microscopy (Figure S8b). The bonded PDMS mold was left on the device to act as an encapsulation layer.

Devices were tested and characterized with source and target electrolyte solutions of 100 mM NaCl(aq) with PEDOT:PSS on polyethylene terephthalate (PET) strips used as replaceable electrodes. Cl^−^ ion transport through the C-HPG5 membrane was achieved by applying a constant current of 100 nA between the PEDOT:PSS electrodes (Figure S9) (voltage was recorded continuously). Assuming perfect permselectivity and using the OEIP's channel geometry and voltage measurements (from 4 OEIP devices tested), the conductivity (ρ) for Cl^−^ ions was calculated using the formula:

(2) $$\begin{array}{l} \rho =\frac{l\times I}{w\times h\times V} \end{array}$$

where *l*, *w*, and *h* are the length, width, and height of the channel, and *I* and *V* the measured current and voltage. An average conductivity value of 0.24 ± 0.02 S m^−1^ for in the C-HPG5 polyelectrolyte channel was calculated (Table 3).

**Table 3:** C-HPG5-based OEIP characterization (100 mM NaCl(aq) as source and target electrolytes).

**Dev**.	**Length (mm)**	**Width (μm)**	**Height (μm)**	**Cross section area (μm^**2**^)**	**Current (nA)**	**Voltage (V)**	**Conductivity (S m^**−1**^)**
1	7.0	85	43	3,655	100	0.73	0.26
2	6.5	85	43	3,655	100	0.75	0.24
3	7.5	85	43	3,655	100	0.85	0.24
4	7.3	85	43	3,655	100	0.89	0.22
Avg.	7.1 ± 0.4						± 0.02

As a demonstration of the C-HPG5 polyelectrolyte's capability to electrophoretically transport larger and more rigid molecular structures, the dye molecule methyl blue^2−^ (10mM(aq), Figure 5B), was used as a source electrolyte. The transport of methyl blue^2−^ was readily observed visually as the blue dye gradually filled the channel (Figure 5C).

## Discussion

The polymer core of hyperbranched polyglycerol (5,000 Da) used in this study was chosen as it has been well-studied since its original synthesis (Sunder et al., 1999), with low polydispersity (Sunder et al., 2000), high degree of branching (“dendrimer-like”) structure (Abbina et al., 2017), biocompatibility (Frey and Haag, 2002; Calderón et al., 2010; Wilms et al., 2010), and thoroughly elucidated structural properties (Baille et al., 2004; Kainthan et al., 2006; Schubert et al., 2016). This versatile material, coupled with the stability of the unreactive ether bonds, allowed the final polyelectrolyte membrane to operate at elevated pH and voltages without structural breakdown. Previous studies have reported hyperbranched polyglycerols with cationic side-group functionalization (Schwab and Mecking, 2005) as well as alkene functionalities for thiol-ene click chemistry reactions (Fu et al., 2008; Killops et al., 2008; Nilsson et al., 2008; Trey et al., 2010). In this study, we demonstrate the possibility of precisely tailoring the properties of the hyperbranched polyglycerols with multi-functionalization by controlled introduction of both charged cationic and allyl groups. This multi-functionalization enabled thiol-ene crosslinking with tri-thiolated polyethylene glycol (Thiocure ETTMP 1300), forming a uniform charged membrane network primarily consisting of branched polyethylene glycol structures. The resulting membranes were then incorporated as the ion channel in OEIPs and demonstrated to be able to sustain selective electrophoretic “ion pumping” in this device configuration.

With the initial step of allylation (Scheme 1), the degree of substitution (DS) was calculated from the ^1^H-NMR spectra (Figure S1a) to ~10 % following addition of 0.2 eq. (to eq. –OH of HPG5) of NaH base followed by addition of 0.2 eq. allylbromide to the HPG5 in DMF solvent. Though the resulting allyl functionalization corresponded to half the amount of added reagents, this was ultimately attributed to the freshness of the NaH reagent used in the synthesis. Initial trial reactions (not included) resulted in a DS ~15–20%. Likewise, a higher degree of allyl functionalization (40–60%) could be achieved in DMF by utilizing more equivalents of NaH and allylbromide reagents. Regardless, the resulting degree of 10% allyl groups was deemed sufficient, and repeated synthesis resulted in overall DS 10–20% with the calculated ^1^H-NMR estimation.

In the proceeding reaction, excess chloroacetyl chloride was used to ensure full conversion of the remaining hydroxyl groups of the (allyl-)HPG5, which could be clearly observed in the ^1^H- and ^13^C-NMR (Figures S2a,b). By ^1^H-NMR integral calculation via α-protons of the chloroacetyl signal compared to HPG5 core protons (Figure S2a), a DS could be estimated to >100% (clearly an overestimation). The overestimation arises due to signal overlapping from the allyl functionality (HPG-O-CH_2_-CH = CH_2_) with new chemical shifts arising from protons corresponding to HPG5 located close to the introduced ester functionalities (HPG-CH-COO-CH_2_Cl and HPG-CH_2_-COO-CH_2_Cl). By calculating the integral of the chloroacetyl α-proton signal integral toward the HPG5 including the allyl functionality signals (estimating 10% DS for 5H), a more realistic DS value of ~90% was obtained.

In the final synthetic step of substituting triphenylphosphine and quaternizating the phosphor into charged phosphonium, the DS was calculated as above, with the triphenyl integral signals toward combining the other shifts, resulting in an estimation of ~60% (Figure S3a). These large functionalization groups increased the molecular weight of the HPG5 considerably, from 5 to ~20 kDa (approximated theoretical molecular weight calculation), and thus dominate the overall signal. An interesting observation in the ^13^C-NMR was the strong signals from the phenyl carbons (Figure S3b), which dominated the intensity and seemed to suppress the rest of the structure's spectra, making it hard to achieve and render a clear resolution for the total structure. With ^31^P-NMR, a distinct shift from the triphenylphosphine reagent signal of −5.6 ppm into a broad quaternized phosphonium signal ~19–20 ppm was observed. Interestingly, an extra peak at 32 ppm was detecting in methanol-d_4_ solution, which was not seen in deuterium oxide solution of the C-HPG5 (Figure S3c). This was attributed to the possibility of solvent-dependent structural conformation of the triphenylphosphonium functionality (Aganova et al., 2014).

In studying the thermal cross-linking of the C-HPG5, various requirements for the success of the reaction were derived. First, it was found that adding a small amount of TATATO was crucial for cross-linking with Thiocure ETTMP 1300. Second, we observed a dependency on high concentration formulation (Table 1), which led to a very swift observable gel formation. A possible explanation could be connected with the survival of radical species for initiation of the reaction with AIBN. The substantial macromolecular sizes of both C-HPG5 polymer and Thiocure 1300 cross-linker alone could have rendered the arrangement of the system too slow for cross-linking radical reactions to take place. The smaller TATATO may diffuse more readily and associate with the macromolecules' functionalities to perform chain-transfer in the polymerization mechanism—quickly enough for the radical species' survival. It was also observed that the equivalents of radical initiator AIBN were not associated with the success of the thermally initiated gel membrane formation, with 6.5 wt% and 0.2 wt% (Figure 3; Table 1, TM1 and TM7). But the AIBN equivalents did affect the pore size formation in the membrane by the evaporation of the low boiling point solvent. This may have been due to the crosslinking density formed and thiol-ene conversion efficiency of AIBN (Uygun et al., 2010) coupled with the solvent incorporation, and possibly related to N_2_-gas evolution from the radical species breakdown.

*In situ* monitoring of thermal cross-linking in DMSO-d_6_ (Figure 4) indicated that the reaction initiated after ~12 min, and proceeded in an ultra-fast (within ~1 min) polymerization with substantial signal intensity loss, ppm shift, and broadening of the entire spectrum. Since the sample was heated at 70°C for thermal initiation of AIBN, DMSO evaporation was not an issue and was thus ruled out as a cause of signal intensity loss. The initiation delay may be related to the presence of oxygen-forming less-reactive radicals (Cook et al., 2008) and/or the requirement for the macromolecular mixture components to arrange in a kinetically favorable orientation, with functionalities associated with each other. The resulting smooth homogenous gels (Figure 2c; Table 1, TD2_DMSO−*d6*_) were attributed to the high boiling point DMSO solvent, high concentration polymer mixture, and low amounts of AIBN initiator.

Free-standing membranes of C-HPG5 (Figure S5) were achieved in a straightforward manner using photo-initiation (Irgacure 2959, curing at 365 nm). The absence of elevated temperature enabled the use of a water/methanol solvent mixture, resulting in pre-hydrated membranes without large solvent evaporation pores. Adjusting various formulation and fabrication parameters with C-HPG5, such as solvent, polymer concentration, and thermal/photo-initiation, allows for a range of customizable hyperbranched polyelectrolyte membranes with specific nanocomposite properties and electrophoretic transport capabilities (Demina et al., 2010). Both the photo- and thermal-initiated gels comprising 70–75% C-HPG5 (excluding solvent content) were able to withstand drying, swelling, and solvent exchange such as methanol and water (Figure S6). These materials displayed soft, flexible characteristics, especially in the dry state, while being more brittle in the fully hydrated state.

One of the general interests in hyperbranched/dendritic polymers is their ability to contain solvents and molecules (Oudshoorn et al., 2006; Wang et al., 2010; Zhang et al., 2013; Pedron et al., 2017). The C-HPG5 polymer, with its large amount of triphenylphosphonium charged functionality, displayed amphiphilic solvating capabilities in a very small amount of solution (80% w/w in methanol), providing the possibility of processing the polymer with low amounts of organic solvents. A possible explanation could be related to structural arrangement, where the compact, spherical shape of hyperbranched macromolecules (Kainthan et al., 2006) makes them amenable to close-packing. By utilizing these covalently-arranged three-dimensional polymer structures coupled with charged surface functionalities, well-defined/tuned cavities in the close-packing between the different macromolecules could provide selective ion transport channels. This leads to our more specific interest in hyperbranched/dendritic polymers: their use as ion transport materials for OEIPs and other iontronic devices (Arbring Sjöström et al., 2018). The C-HPG5 polymer was thus demonstrated as the ion transport channel in an OEIP system, with a specific formulation chosen to achieve molding via the MIMIC technique (Table 2, UVC4, Figure S8). Stable electrophoretic transport of Cl^−^ was observed in the C-HPG5 channels, resulting in an average ionic conductivity of 0.24 ± 0.02 S/m. By employing methyl blue at the source, ionic exchange and transportation of the large anionic (799.81 g/mol) dye molecule were accomplished through the cationic hyperbranched polyelectrolyte membrane.

In conclusion, demands for new ionically charged organic materials capable of versatile manufacturing processing, selective ion transport including large/aromatic compounds, and biocompatibility sets increasing requirement on the polyelectrolytes used in organic (bio)electronics. We have synthesized a new type of multi-functionalized hyperbranched polyglycerol with cationic charges and functionalities for click chemistry cross-linking (thermal- and photo-initiated), to form monolithic membranes. The membrane network formation reaction was investigated, which by initiation proceeded in an ultra-rapid fashion to form insoluble gels capable of solvent exchange and large anionic electrophoretic transportation. The amphiphilic nature of the polyelectrolyte allows for simple implementation to create pre-formed hydrated/loaded membranes applied directly into devices.

## Materials and Methods

Hyperbranched polyglycerol used with molecular weight corresponding to 5,000 Da and degree of branching 53.6%, based on GPC and inverse gated ^13^C-NMR, respectively, was purchased from NanoPartica GmbH. Thiocure® ETTMP 1300 was obtained from Bruno Bock Chemische. All other chemicals were purchased from Merck-Sigma Aldrich with highest purity and used as received. Dialysis tubing Spectra/Por^©^ Regenerated Cellulose with molecular weight cut-off (MWCO) were bought from Spectrum Laboratories. Synthesis reactions were carried out in nitrogen atmosphere, with glassware dried in a 110°C oven. Reaction solvents DMF and chloroform were pre-dried (>16 h) with 4 Å molecular sieves and deaerated with nitrogen (30–60 min) prior to use. For the functionalization of hyperbranched polyglycerol 5,000 Da, reagent equivalents were calculated and used corresponding to relative hydroxyl functionality (67.5). ^1^H-, ^13^C- and ^31^P-NMR analysis were recorded at 300, 75, and 121 MHz, respectively, on a Varian Oxford 300 MHz spectrometer using internal solvent peaks as reference (Gottlieb et al., 1997). NMR FID files were processed with MestReNova v12.04-22023 (Mestrelab Research S.L.).

### Allyl-Hyperbranched Polyglycerol (Compound 2)

Hyperbranched polyglycerol (HPG, 5,000 Da, Compound 1) (2 g, 27.00 mmol –hydroxyl functionality) was dissolved in dry DMF (54 mL) followed by addition of sodium hydride (60% oil dispersion, 216 mg, 5.40 mmol, 0.2 equivalents) and was left to stir for 2 h under nitrogen atmosphere at room temperature. Allyl bromide (468 μL, 5.40 mmol, 0.2 equivalents) was added and the reaction was left overnight (16 h) until it was quenched with ice chips. The solvent was reduced via rotary evaporation and the residuals were re-dissolved in H_2_O (60 mL), filtered with a 0.45 μm PVDF membrane, washed twice with ethyl acetate (2 × 60 mL). Dialysis in H_2_O was then performed with a regenerated cellulose (3,500 Da MWCO) membrane. Water was removed by rotary evaporation and the product was placed *in vacuo* to yield a transparent white/yellow viscous gel (Compound 2).

^1^H NMR (300 MHz, Deuterium Oxide) δ: 6.17–5.94 (m, 1H, -OCH_2_CH = CH_2_), 5.53–5.29 (m, 2H, -OCH_2_CH = CH_2_), 4.32–4.12 (m, 2H, -OCH_2_CH = CH_2_), 4.12–3.38 (m, 5H, dPG), 1.55–1.36 (m, 2H, TMP_core_ -CH_2_CH_3_), 1.03–0.88 (m, 3H, TMP_core_ -CH_2_CH_3_).

^1^H NMR (300 MHz, DMSO-*d*_6_) δ: 6.03–5.75 (m, 1H, -OCH_2_CH = CH_2_), 5.35–5.02 (m, 2H, -OCH_2_CH = CH_2_), 4.94–4.40 (HO-HPG), 4.14–3.86 (m, 2H, -OCH_2_CH = CH_2_), 3.86–2.87 (m, 5H, dPG), 1.37–1.16 (m, 2H, TMP_core_ -CH_2_CH_3_), 0.88–0.69 (m, 3H, TMP_core_ -CH_2_CH_3_).

^13^C NMR (75 MHz, DMSO-*d*_6_) δ: 135.88, 135.51, 116.61, 116.25, 80.00, 78.11, 72.99, 71.56, 71.03, 70.58, 69.65, 69.01, 68.73, 63.13, 61.09.

### Chloroacetate-Allyl-Hyperbranched Polyglycerol (Compound 3)

Allyl-HPG5 (Compound 2) (1,108 mg, 13.50 mmol) was dissolved in dry DMF (27 mL) and cooled with an ice bath. A solution of chloroacetyl chloride (2.68 mL, 33.75 mmol, 2.5 eq.) in dry DMF (18 mL) was prepared and added drop-wise to the polymer solution under stirring and nitrogen atmosphere, followed by slow drop-wise addition of triethylamine (1.88 mL, 13.50 mmol, 1 eq.). The reaction was left to stir for 16 h at room temperature, quenched with ice chips, and filtered through a 0.45 μm PVDF membrane. Solvent volume was reduced via evaporation and the residuals were dissolved in chloroform (50 mL). The organic phase was washed with H_2_O (50 mL), brine (50 mL), and H_2_O (2 × 50 mL), and dried with MgSO_4_. The salts were filtered off, the chloroform was removed via rotary evaporation, and the product was placed *in vacuo* to yield a brown viscous oil (Compound 3).

^1^H NMR (300 MHz, Chloroform-*d*) δ: 5.96–5.76 (m, 1H, -OCH_2_CH = CH_2_), 5.31–5.02 (m, 2H, -OCH_2_CH = CH_2_), 4.52-4.22 (m, 3H, HPG-CHCOO-CH_2_Cl, HPG-CH_2_COO-CH_2_Cl), 4.14–4.03 (m, 2H, -COOCH_2_Cl), 4.21–3.91 (m, 2H, -OCH_2_CH = CH_2_), 3.87–3.19 (m, 5H, hPG), 1.50–1.26 (m, 2H, TMP_core_ -CH_2_CH_3_), 0.92–0.74 (m, 3H, TMP_core_ -CH_2_CH_3_);

^13^C NMR (75 MHz, Chloroform-*d*) δ 167.37, 167.09, 166.94, 166.85, 135.29, 134.66, 117.45, 116.94, 78.89, 77.58, 77.16, 76.74, 73.11, 72.27, 71.73, 71.33, 70.60, 69.91, 69.26, 69.13, 68.28, 66.79, 65.26, 64.04, 63.83, 41.05, 40.84, 40.68.

### Triphenylphosphoniumacetate-Allyl-Hyperbranched Polyglycerol Chloride (Compound 4, C-HPG5)

Chloroacetate-allyl-HPG5 (Compound 3) (1.32 mg, 9.00 mmol) was dissolved in chloroform (18 mL) together with triphenylphosphine (4.73 g, 18.02 mmol, 2 eq.) in a microtube that was sealed with a cap and purged with N_2_-gas. The reaction mixture was left to stir for 16 h at 60°C after which the solvent was removed by evaporation. Remaining crude product was dissolved in a minimum amount of methanol and trituration were performed by pouring cold diethyl ether to precipitate the product. The diethyl ether was decanted and the procedure was repeated four times after which remaining solvents were removed with rotary evaporation. The remaining product was dissolved in water and dialysis in the medium with a regenerated cellulose membrane (3,500 Da MWCO) was performed. Azeotropic evaporation with toluene (2 × 20 mL) were performed and the remains were placed *in vacuo* to yield a solid yellow crystalline product (Compound 4, C-HPG5).

^1^H NMR (300 MHz, Methanol-*d*_4_) δ: 8.22–7.45 (m, 15H, -P^+^Ph_3_), 6.00–5.71 (m, 1H, -OCH_2_CH = CH_2_), 5.33–4.90 (m, 2H, -OCH_2_CH = CH_2_; m, 1H, HPG-CHCOO-), 4.35–3.86 (m, 2H,-OCH_2_CH = CH_2_; m, 2H, HPG-CH_2_COO-), 3.84–2.91 (m, 2H, -COOCH_2_P^+^Ph_3;_ m, 5H, HPG).

^13^C NMR (75 MHz, Methanol-*d*_4_) δ: 165.98, 136.57, 135.15, 135.01, 133.73, 133.11, 132.98, 131.58, 131.41, 130.04, 129.88, 120.00, 118.81, 117.44, 117.19, 79.87, 74.04, 73.27, 72.19, 71.17, 68.97, 42.17, 30.86.

^31^P NMR (121 MHz, Methanol-*d*_4_) δ 32.16, 20.64–19.56 (m).

^31^P NMR (121 MHz, Deuterium Oxide) δ: 19.94–19.06 (m).

### Thermal Cross-Linking Gel

C-HPG5 (400 mg, 1.135 mmol, Compound 4) was dissolved in methanol in a vial (200 μL) and mixed together with Thiocure 1300 ETTMP (400 mg, 0.303 mmol), Triallyl-1,3,5-triazine-2,4,6(1H,3H,5H)-trione (21 mg, 0.084 mmol), and 2,2'-Azobis(2-methylpropionitrile) (2 mg, 0.012 mmol) by vortexing. The vial was deaerated, sealed and heated to 75 °C overnight into an insoluble continuous gel.

### Thermal Cross-Linking Gel for NMR

C-HPG-5 (341 mg, Compound 4) was added into a vial and dissolved in DMSO-d_6_ (300 μL) by vortexing for 5 min. This was followed by addition of Thiocure 1300 ETTMP (89 mg), triallyl-1,3,5-triazine-2,4,6(1H,3H,5H)-trione (18 mg, 0.072 mmol), and 2,2′-Azobis(2-methylpropionitrile) (17 mg, 0.104 mmol) which was vortexed an additional 5 min into a homogenous viscous solution. The mixture was transferred into an NMR-tube, deaerated with N_2_-gas, sealed with a cap, and heated for 15 min at 70°C inside a Varian Oxford 300 MHz spectrometer, where the reaction was monitored by acquisition of a ^1^H-NMR spectra collected every minute.

### Organic Electronic Ion Pump Fabrication

#### PDMS Master Mold Fabrication

SU-8 3050 (MicroChem Corp.) was spin-coated (5 s spread at 500 rpm, 30 s spin at 3,000 rpm) on a 4 silicon wafer and soft baked on a leveled hot plate (1 min hold at 65°C, increasing with 10°C/min to 95°C, and hold for 15 min). After cooling, the wafer was exposed through a plastic mask with 300 mJ/cm^2^ (measured at 365 nm) using a Karl Suss MA/BM 6 mask aligner equipped with an I-line filter. The exposed parts were cross-linked by ramped hard bake (as soft bake, but final hold at 95 °C for 5 min) and developed using two baths of mr-Dev 600 (Micro resist technology GmbH), each for 5 min, followed by a 10 s rinse in isopropanol and drying using N_2_. The final SU-8 pattern was additionally hard baked at 110°C for 5 min.

#### PDMS Master Mold Characterization

The width and depth of the SU-8 pattern was measured with an optical profilometer (Sensofar PLu Neox) using a 50x confocal objective.

#### PDMS Mold

PDMS (DowSil Sylgard 184) was prepared at a 10:1 base/curing agent ratio, and mixed and degassed. The blend was poured on the PDMS master mold to a thickness of ~2 mm and cured on a hot plate (1 h at 120°C). The PDMS mold was carefully peeled off the master, and cut into pieces, with each piece containing a capillary channel connecting two electrolyte reservoirs.

#### Channel Filling

Glass wafers were cleaned using Hellmanex^®^ III, rinsed, and baked prior to use. Glass wafers and PDMS pieces were O_2_-plasma treated (100 W at 800 mTorr for 5 min) and subsequently immediately bonded together. C-HPG5 (50 mg, Compound 4), Thiocure 1300 ETTMP (13 mg, 0.01 mmol), 1,3,5-triallyl-1,3,5-triazine-2,4,6(1H,3H,5H)-trione (2.6 mg, 0.010 mmol), and 2-hydroxy-4′-(2-hydroxyethoxy)-2-methylpropiophenone (3.4 mg, 0.015 mmol) were dissolved in methanol (50 μL) and water (50 μL). The solution was vortexed for 2 min and the channels were filled by capillary action by placing 1 μL of membrane solution at one end. The polyelectrolyte was cross-linked for 10 min in a Karl Suss MA/BM 6 mask aligner (flood exposure, 10 mJ/cm^2^ measured at 365 nm) under nitrogen atmosphere. The channels were then soaked in 0.1 mM KCl solution by placing droplets at the inlet and outlet for 3 h.

#### Scanning Electron Microscopy Imaging

Channels were prepared as above but on a silicon wafer with 50 nm gold deposited by thermal evaporation. Further, to enable removal of the mold after filling and AEM crosslinking, neither the substrate or PDMS mold were activated by plasma. Finally, a 5 nm layer of gold was evaporated on top of the AEM. Images were acquired using a Zeiss Sigma 500 Gemini scanning electron microscope at 2 kV acceleration voltage and with the in-lens secondary electron detector.

#### Channel Characterization

The conductivity of C-HPG5-based OEIPs were determined by placing PEDOT:PSS (coated on polyethylene terephthalate) electrodes in 0.1 M NaCl(aq) electrolytes on both side of the channel, applying a current of 100 nA, and measuring the required driving voltage using a Keithley Source Meter 2612 (Tektronix, Inc.). To assess the transport of larger molecules, the source solution was exchanged to the dye molecule methyl blue^2−^ (10 mM aqueous).

## Data Availability

The datasets generated for this study are available on request to the corresponding author.

## Author Contributions

TA performed all synthesis and chemical characterization. DP and EG fabricated and characterized OEIP devices. MS advised and planned synthesis and chemical characterization. DS, MB, and MS conceived of the project. TA wrote the manuscript, with assistance from EG, MS, and DS.

### Conflict of Interest Statement

MS is employed by the independent state-owned research institute RISE Acreo AB. DP, DS, MB, and TA are shareholders in the small, researcher-controlled intellectual property company OBOE IPR AB (oboeipr.com), which owns the patents related to this research. The remaining author (EG) declares that the research was conducted in the absence of any commercial or financial relationships that could be construed as a potential conflict of interest.

## References

[B1] Abbina S. Vappala S. Kumar P. Siren E. M. J. La C. C. Abbasi U. (2017). Materials chemistry B hyperbranched polyglycerols: recent advances in synthesis, biocompatibility and biomedical applications. J. Mater. Chem. B 5, 9241–9420. 10.1039/C7TB02515G 32264530

[B2] Abidian M. R. Kim D. H. Martin D. C. (2006). Conducting-polymer nanotubes for controlled drug release. Adv. Mater. 18, 405–409. 10.1002/adma.200501726 21552389 PMC3088882

[B3] Aganova O. Galiullina L. Aganov A. Shtyrlin Y. Pugachev M. Shtyrlin N. (2014). The study of the conformation and dynamics of the new quaternary phosphonium salts by NMR spectroscopy. Appl. Magn. Reson. 45, 653–665. 10.1007/s00723-014-0544-4

[B4] Arbring Sjöström T. Berggren M. Gabrielsson E. O. Janson P. Poxson D. J. Seitanidou M. (2018). A decade of iontronic delivery devices. Adv. Mater. Technol. 3:1700360 10.1002/admt.201700360

[B5] Baille W. E. Zhu X. X. Fomine S. (2004). Study of self-diffusion of hyperbranched polyglycidols in poly(vinyl alcohol) solutions and gels by pulsed-field gradient NMR spectroscopy. Macromolecules 37, 8569–8576. 10.1021/ma049588a

[B6] Berggren M. Richter-Dahlfors A. (2007). Organic bioelectronics. Adv. Mater. 19, 3201–3213. 10.1002/adma.200700419

[B7] Burakowska E. Haag R. (2009). Dendritic polyglycerol core-double-shell architectures: synthesis and transport properties. Macromolecules 42, 5545–5550. 10.1021/ma9005044

[B8] Calderón M. Quadir M. A. Sharma S. K. Haag R. (2010). Dendritic polyglycerols for biomedical applications. Adv. Mater. 22, 190–218. 10.1002/adma.200902144 20217684

[B9] Cook W. D. Chen F. Pattison D. W. Hopson P. Beaujon M. (2008). Thermal polymerization of thiol–ene network-forming systems Wayne. Polym. Int. 57, 171–180. 10.1002/pi.2314

[B10] Demina O. A. Demin A. V. Gnusin N. P. Zabolotskii V. I. (2010). Effect of an aprotic solvent on the properties and structure of ion-exchange membranes. Polym. Sci. Ser. A 52, 1270–1282. 10.1134/S0965545X10120059

[B11] Frey H. Haag R. (2002). Dendritic polyglycerol : a new versatile biocompatible material. Rev. Mol. Biotechnol. 90, 257–267. 10.1016/S1389-0352(01)00063-0 12071228

[B12] Fu Q. Liu J. Shi W. (2008). Preparation and photopolymerization behavior of multifunctional thiol–ene systems based on hyperbranched aliphatic polyesters. Prog. Org. Coatings 63, 100–109. 10.1016/j.porgcoat.2008.04.014

[B13] Gode P. Hult A. Jannasch P. Johansson M. Karlsson L. Lindbergh G. (2006). A novel sulfonated dendritic polymer as the acidic component in proton conducting membranes. Solid State Ionics 177, 787–794. 10.1016/j.ssi.2005.12.031

[B14] Gottlieb H. E. Kotlyar V. Nudelman A. (1997). NMR chemical shifts of common laboratory solvents as trace impurities. J. Org. Chem. 62, 7512–7515. 10.1021/jo971176v 11671879

[B15] Hoyle C. E. Bowman C. N. (2010). Thiol-ene click chemistry. Angew. Chem. Int. Ed. 49, 1540–1573. 10.1002/anie.200903924 20166107

[B16] Isaksson J. Kjäll P. Nilsson D. Robinson N. Berggren M. Richter-Dahlfors A. (2007). Electronic control of Ca^2+^ signalling in neuronal cells using an organic electronic ion pump. Nat. Mater. 6, 673–679. 10.1038/nmat1963 17643105

[B17] Jonsson A. Inal S. Uguz I. Williamson A. J. Kergoat L. Rivnay J.. (2016). Bioelectronic neural pixel: Chemical stimulation and electrical sensing at the same site. Proc. Natl. Acad. Sci. U.S.A. 113, 9440–9445. 10.1073/pnas.1604231113 27506784 PMC5003234

[B18] Jonsson A. Song Z. Nilsson D. Meyerson B. A. Simon D. T. Linderoth B.. (2015). Therapy using implanted organic bioelectronics. Sci. Adv. 1:e1500039. 10.1126/sciadv.1500039 26601181 PMC4640645

[B19] Kainthan R. K. Muliawan E. B. Hatzikiriakos S. G. Brooks D. E. (2006). Synthesis, characterization, and viscoelastic properties of high molecular weight hyperbranched polyglycerols. Macromolecules 39, 7708–7717. 10.1021/ma0613483

[B20] Kasprzak S. E. Martin B. Raj T. Gall K. (2009). Synthesis and thermomechanical behavior of (qua)ternary thiol-ene(/acrylate) copolymers. Polymer 50, 5549–5558. 10.1016/j.polymer.2009.09.044 22973067 PMC3437513

[B21] Killops K. L. Campos L. M. Hawker C. J. (2008). Robust, efficient, and orthogonal synthesis of dendrimers via thiol-ene “click” chemistry. J. Am. Chem. Soc. 130, 5062–5064. 10.1021/ja8006325 18355008

[B22] Kim E. Xia Y. Whitesides G. M. (1996). Micromolding in capillaries: applications in materials science. J. Am. Chem. Soc. 118, 5722–5731. 10.1021/ja960151v

[B23] Kłos J. S. Sommer J.-U. (2010). Simulations of terminally charged dendrimers with flexible spacer chains and explicit counterions. Macromolecules 43, 4418–4427. 10.1021/ma1003997

[B24] Kontturi K. Murtomäki L. Manzanares J. A. J. A. (2008). Chapter 4: Ionic transport processes, in Electrochemistry and Membrane Science (Oxford: Oxford University Press), 126–231. 10.1093/acprof:oso/9780199533817.001.0001

[B25] Kontturi K. Pentti P. Sundholm G. (1998). Polypyrrole as a model membrane for drug delivery. J. Electroanal. Chem. 453, 231–238. 10.1016/S0022-0728(98)00246-0

[B26] Matveeva E. D. Podrugina T. A. Grishin Y. K. Tkachev V. V. Zhdankin V. V. Aldoshin S. M. (2003). Synthesis and structure of mixed phosphonium-iodonium ylide. Russ. J. Org. Chem. 39, 536–541. 10.1023/A:1026011902365

[B27] Nilsson C. Simpson N. Malkoch M. Johansson M. Malmström E. (2008). Synthesis and thiol-ene photopolymerization of allyl-ether functionalized dendrimers. J. Polym. Sci. A Polym. Chem. 46, 1339–1348. 10.1002/pola.22474

[B28] Oudshoorn M. H. M. Rissmann R. Bouwstra J. A. Hennink W. E. (2006). Synthesis and characterization of hyperbranched polyglycerol hydrogels. Biomaterials 27, 5471–5479. 10.1016/j.biomaterials.2006.06.030 16859743

[B29] Pedron S. Pritchard A. M. Vincil G. A. Andrade B. Zimmerman S. C. Harley B. A. C. (2017). Patterning three-dimensional hydrogel microenvironments using hyperbranched polyglycerols for independent control of mesh size and stiffness. Biomacromolecules 18, 1393–1400. 10.1021/acs.biomac.7b00118 28245360 PMC5444810

[B30] Poxson D. J. Gabrielsson E. O. Bonisoli A. Linderhed U. Abrahamsson T. Matthiesen I.. (2019). Capillary fiber-based electrophoretic delivery device. ACS Appl. Mater. Interfaces 11, 14200–14207. 10.1021/acsami.8b22680 30916937

[B31] Proctor C. M. Slézia A. Kaszas A. Ghestem A. del Agua I. Pappa A.-M.. (2018). Electrophoretic drug delivery for seizure control. Sci. Adv. 4:eaau1291. 10.1126/sciadv.aau1291 30167463 PMC6114990

[B32] Schubert C. Osterwinter C. Tonhauser C. Schömer M. Wilms D. Frey H. (2016). Can hyperbranched polymers entangle? Effect of hydrogen bonding on entanglement transition and thermorheological properties of hyperbranched polyglycerol melts. Macromolecules 49, 8722–8737. 10.1021/acs.macromol.6b00674

[B33] Schwab E. Mecking S. (2005). Synthesis and properties of highly branched polycations with an aliphatic polyether scaffold. J. Polym. Sci. A Polym. Chem. 43, 4609–4617. 10.1002/pola.20983

[B34] Seitanidou M. Franco-Gonzalez J. F. Sjöström T. A. Zozoulenko I. Berggren M. Simon D. T. (2017). pH dependence of γ-aminobutyric acid iontronic transport. J. Phys. Chem. B 121, 7284–7289. 10.1021/acs.jpcb.7b05218 28741949

[B35] Seitanidou M. Tybrandt K. Berggren M. Simon D. T. (2019). Overcoming transport limitations in miniaturized electrophoretic delivery devices. Lab. Chip 19, 1427–1435. 10.1039/C9LC00038K 30875418

[B36] Simon D. T. Kurup S. Larsson K. C. Hori R. Tybrandt K. Goiny M.. (2009). Organic electronics for precise delivery of neurotransmitters to modulate mammalian sensory function. Nat. Mater. 8, 742–746. 10.1038/nmat2494 19578335

[B37] Someya T. Bao Z. Malliaras G. G. (2016). The rise of plastic bioelectronics. Nature 540, 379–385. 10.1038/nature21004 27974769

[B38] Sunder A. Frey H. Mülhaupt R. (2000). Hyperbranched polyglycerols by ring-opening multibranching polymerization. Macromol. Symp. 153, 187–196. 10.1002/1521-3900(200003)153:1<187::AID-MASY187>3.0.CO;2-I

[B39] Sunder A. Hanselmann R. Frey H. Mülhaupt R. (1999). Controlled synthesis of hyperbranched polyglycerols by ring-opening multibranching polymerization. Macromolecules 32, 4240–4246. 10.1021/ma990090w

[B40] Thota B. N. S. Urner L. H. Haag R. (2016). Supramolecular architectures of dendritic amphiphiles in water. Chem. Rev. 116, 2079–2102. 10.1021/acs.chemrev.5b00417 26669418

[B41] Trey S. M. Nilsson C. Malmström E. Johansson M. (2010). Thiol-ene networks and reactive surfaces via photoinduced polymerization of allyl ether functional hyperbranched polymers. Prog. Org. Coatings 67, 348–355. 10.1016/j.porgcoat.2009.10.023

[B42] Türk H. Shukla A. Rodrigues P. C. A. Rehage H. Haag R. (2007). Water-soluble dendritic core-shell-type architectures based on polyglycerol for solubilization of hydrophobic drugs. Chem. A Eur. J. 13, 4187–4196. 10.1002/chem.200601337 17310496

[B43] Tybrandt K. Larsson K. C. Kurup S. Simon D. T. Kjäll P. Isaksson J. (2009). Translating electronic currents to precise acetylcholine-induced neuronal signaling using an organic electrophoretic delivery device. Adv. Mater. 21, 4442–4446. 10.1002/adma.200900187

[B44] Uygun M. Tasdelen M. A. Yagci Y. (2010). Influence of type of initiation on thiol-ene “click” chemistry. Macromol. Chem. Phys. 211, 103–110. 10.1002/macp.200900442

[B45] Wadhwa R. Lagenaur C. F. Cui X. T. (2006). Electrochemically controlled release of dexamethasone from conducting polymer polypyrrole coated electrode. J. Control. Release 110, 531–541. 10.1016/j.jconrel.2005.10.027 16360955

[B46] Wang Q. Mynar J. L. Yoshida M. Lee E. Lee M. Okuro K.. (2010). High-water-content mouldable hydrogels by mixing clay and a dendritic molecular binder. Nature 463, 339–343. 10.1038/nature08693 20090750

[B47] Wei Y. (1998). Molecular electronics–the future of bioelectronics. Supramol. Sci. 5, 723–731. 10.1016/S0968-5677(98)00112-6

[B48] Williamson A. J. Rivnay J. Kergoat L. Jonsson A. Inal S. Uguz I.. (2015). Controlling epileptiform activity with organic electronic ion pumps. Adv. Mater. 27, 3138–3144. 10.1002/adma.201500482 25866154

[B49] Wilms D. Stiriba S.-E. Frey H. (2010). Hyperbranched polyglycerols: from the controlled synthesis of biocompatible polyether polyols to multipurpose applications. Acc. Chem. Res. 43, 129–141. 10.1021/ar900158p 19785402

[B50] Zhang H. Patel A. Gaharwar A. K. Mihaila S. M. Iviglia G. Mukundan S.. (2013). Hyperbranched polyester hydrogels with controlled drug release and cell adhesion properties. Biomacromolecules 14, 1299–1310. 10.1021/bm301825q 23394067 PMC3653976

