# Supplementary material for: Formation of Monolithic Ion-Selective Transport Media Based on “Click” Cross-Linked Hyperbranched Polyglycerol
Source: Front Chem. 2019 Jul 10;7:484. doi: 10.3389/fchem.2019.00484 (PMC6635471; doi:10.3389/fchem.2019.00484)
Supplement: Supplementary file 1 [file Data_Sheet_1.PDF]

Supplementary Material

Formation of monolithic ion-selective transport media in pre-formed microfluidic channels

Tobias Abrahamsson[1], David J. Poxson[1], Erik O. Gabrielsson[1], Mats Sandberg[2], Daniel T. Simon[1*], Magnus Berggren[1]

Laboratory of Organic Electronics, Department of Science and Technology, Linköping University, 601 74 Norrköping, Sweden

[2] RISE Acreo AB, 601 17 Norrköping, Sweden

*** Correspondence:** daniel.simon@liu.se

1 Supplementary Data

^{1}H-, ^{13}C- & ^{31}P-NMR characterization and degree of substitution calculation.

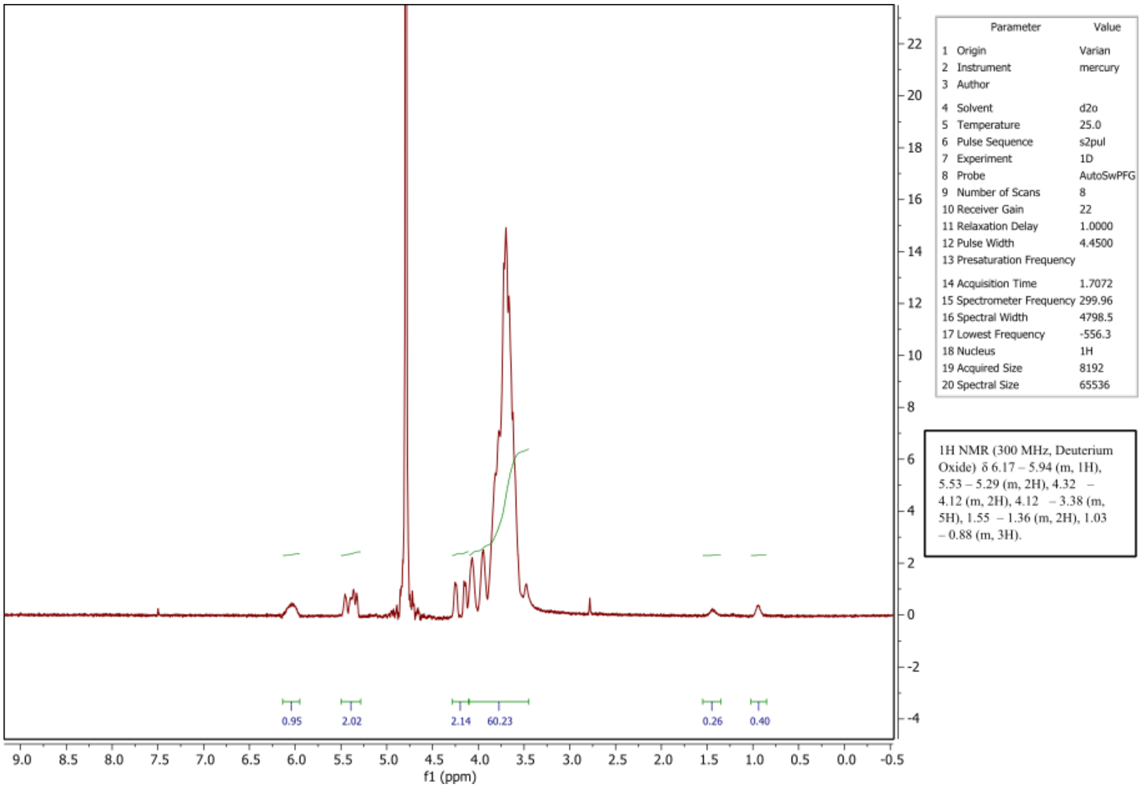

Figure S1a. ^{1}H-NMR (300 MHz, Deuterium Oxide) - Allyl-HPG5 (compound 2).

$$DS\ (\%)\ Allyl = \left(\frac{\frac{2.02}{60.23}}{\frac{2}{5}} \right) x\ 100\ \approx 10\ \%$$

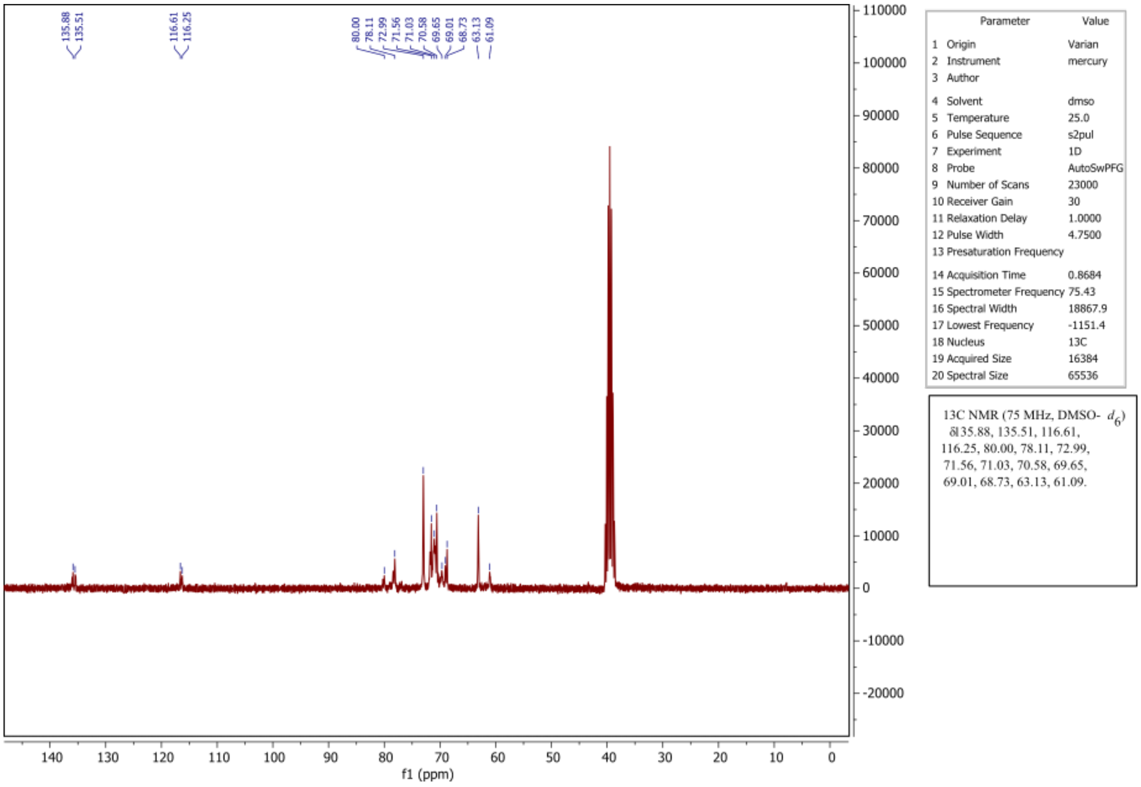

Figure S1b. ^{13}C-NMR (75 MHz, DMSO-d_6) – Allyl-HPG5 (compound 2).

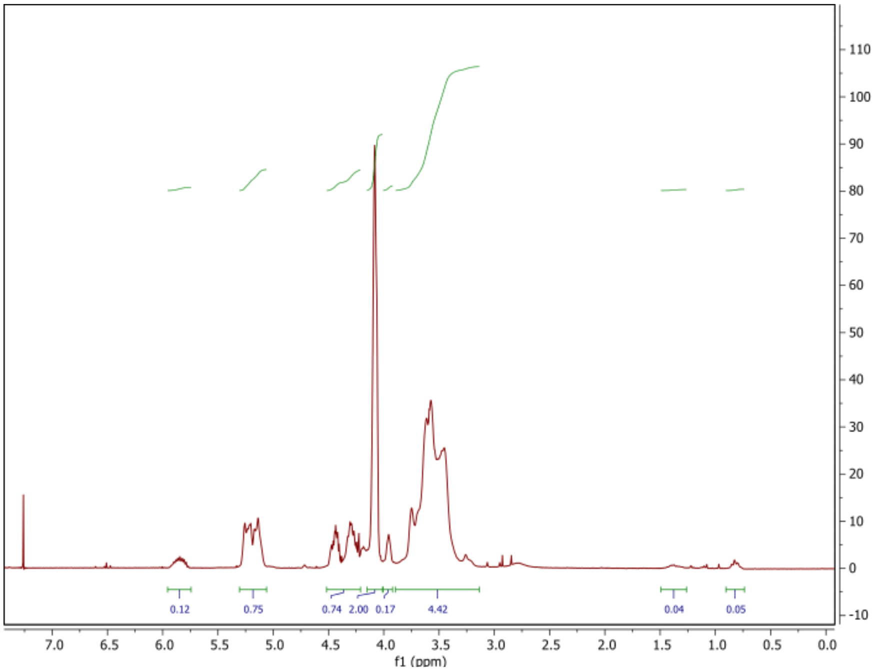

	Parameter	Value
1	Origin	Varian
2	Instrument	mercury
3	Author	
4	Solvent	cdcl3
5	Temperature	25.0
6	Pulse Sequence	s2pul
7	Experiment	1D
8	Probe	AutoSwPFG
9	Number of Scans	8
10	Receiver Gain	16
11	Relaxation Delay	1.0000
12	Pulse Width	4.4500
13	Presaturation Frequency	
14	Acquisition Time	1.7072
15	Spectrometer Frequency	299.96
16	Spectral Width	4798.5
17	Lowest Frequency	-598.7
18	Nucleus	1H
19	Acquired Size	8192
20	Spectral Size	65536

1H NMR (300 MHz, Chloroform-*d*) δ 5.95 – 5.76 (m, 1H), 5.31 – 5.02 (m, 2H), 4.52 – 4.22 (m, 3H), 4.14 – 4.03 (m, 2H), 4.21 – 3.91 (m, 2H), 3.87 – 3.19 (m, 5H), 1.50 – 1.26 (m, 2H), 0.92 – 0.74 (m, 3H).

Figure S2a. ^{1}H-NMR (300 MHz, Chloroform-*d*) – Chloroacetate-allyl-HPG5 (compound 3).

$$DS\ (\%) Chloroacetate = \left(\frac{\frac{2}{\frac{4.42 + 0.17 + 0.74 + 0.75 + 0.12}{2}}}{5 + 5 * 0.1} \right) x\ 100\ \approx 90\ \%$$

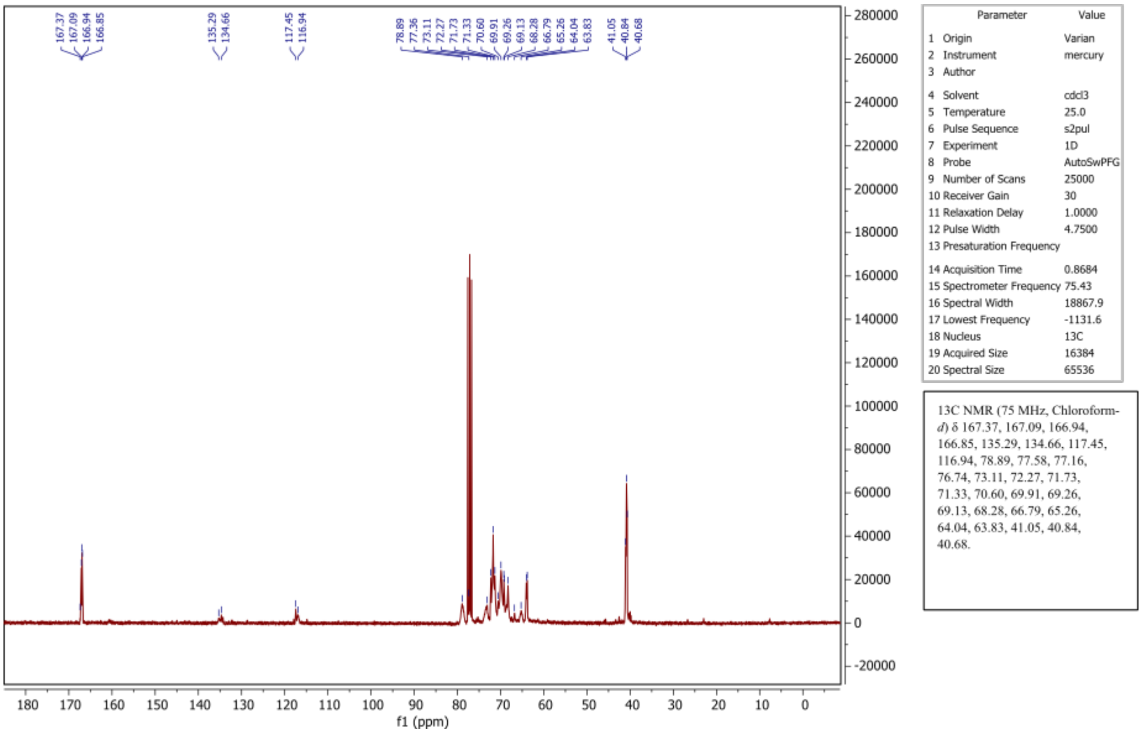

Figure S2b. ^{13}C-NMR (75 MHz, Chloroform-*d*) - Chloroacetate-allyl-HPG5 (compound 3).

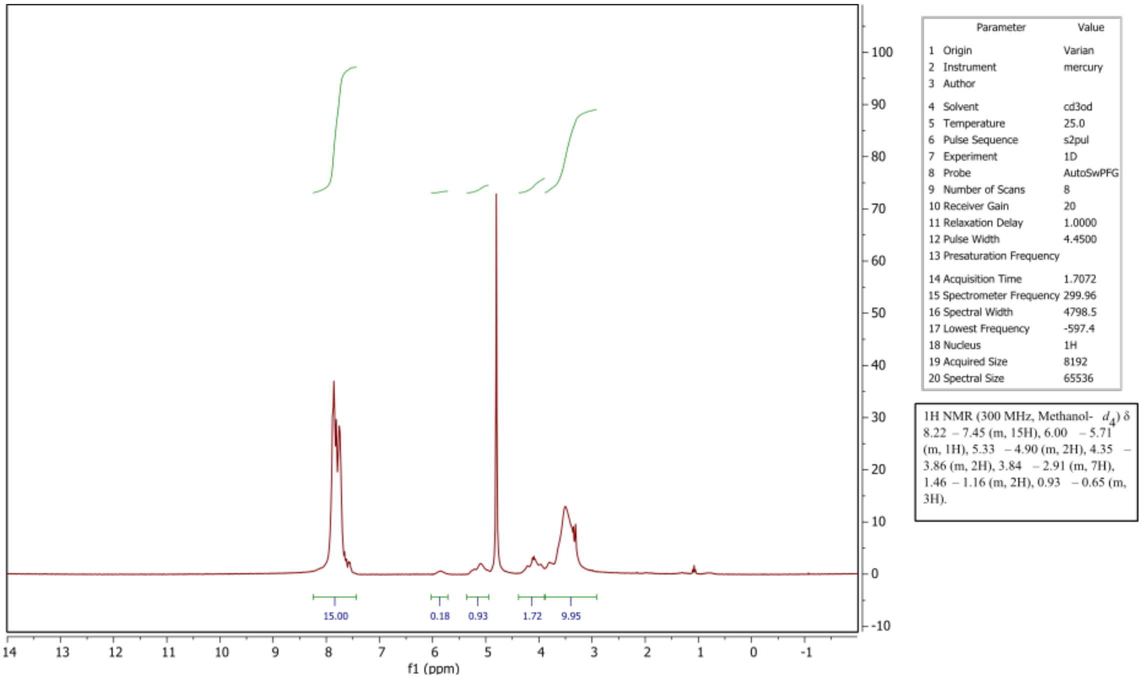

Figure S3a. ^{1}H NMR (300 MHz, Methanol-d_4) – C-HPG5 (compound 4).

$$DS\ (\%) Triphenylphosphonium = \left(\frac{\frac{15}{9.95 + 1.72 + 0.93 + 0,18}}{\frac{15}{5 + 5 * 0.1 + 2 * 0,9}} \right) x\ 100\ \approx 60\ \%$$

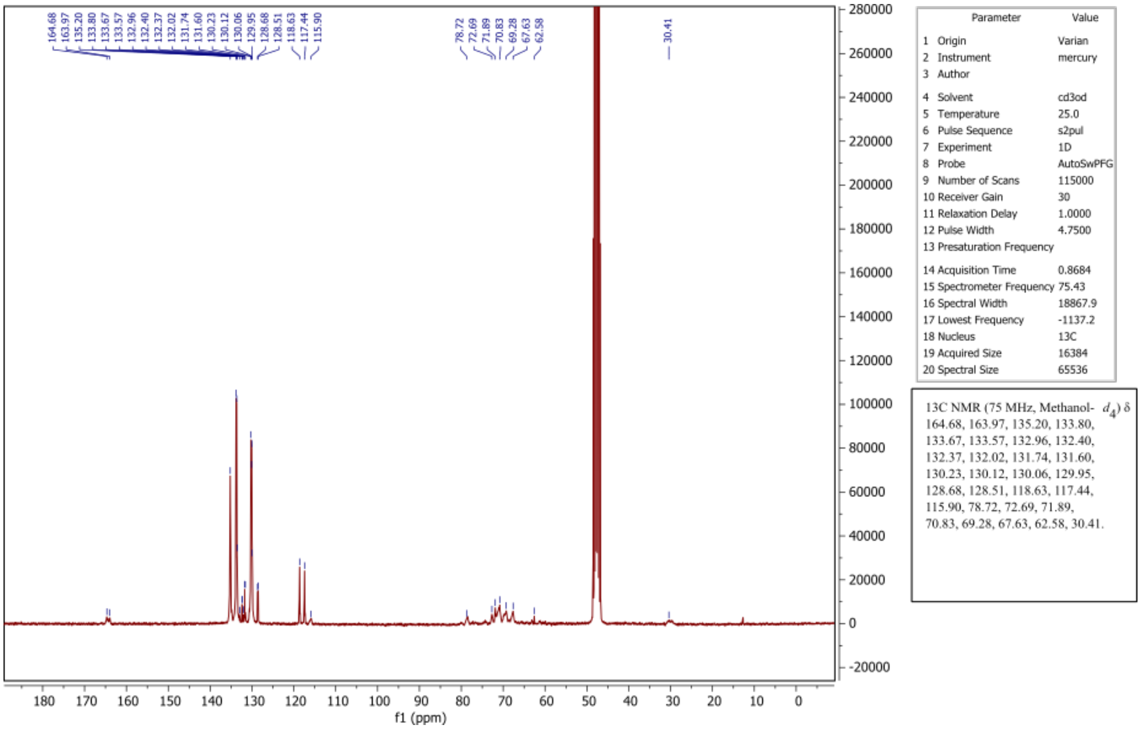

Figure S3b. ^{13}C NMR (75 MHz, Methanol-d_4) – C-HPG5 (compound 4).

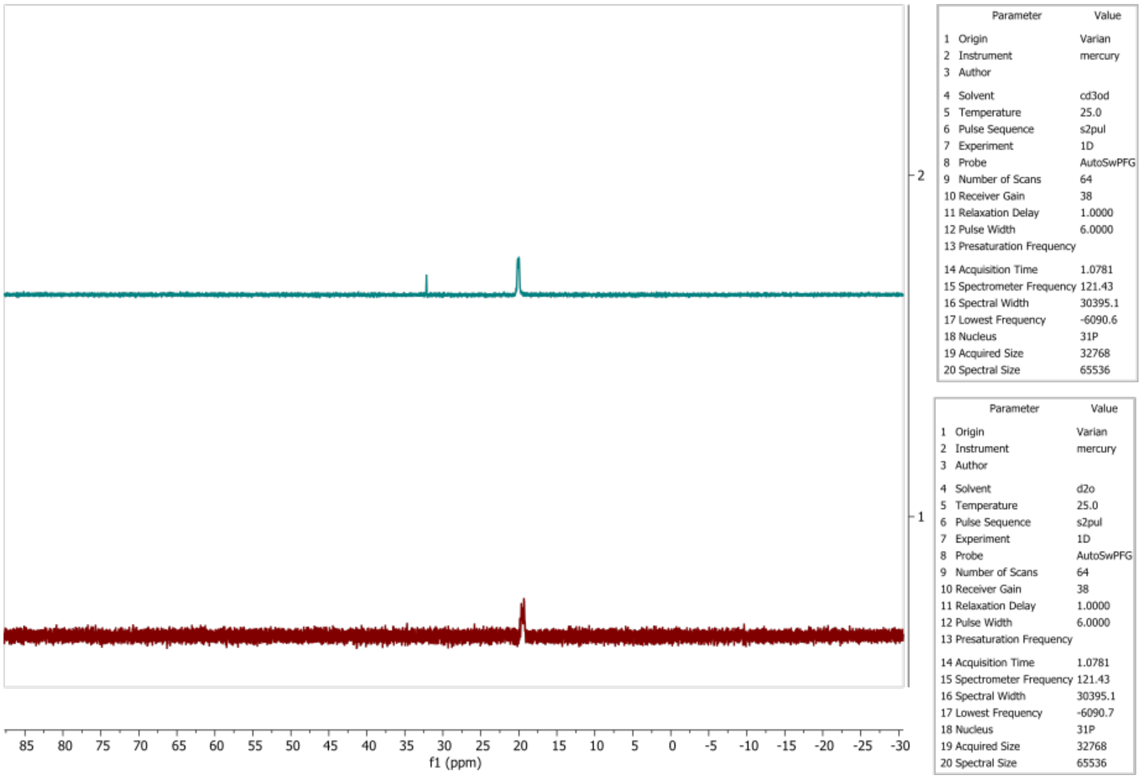

Figure S3c. ^{31}P NMR (121 MHz,Top: Methanol-d_4, Bottom: Deuterium Oxide) – C-HPG5 (compound 4).

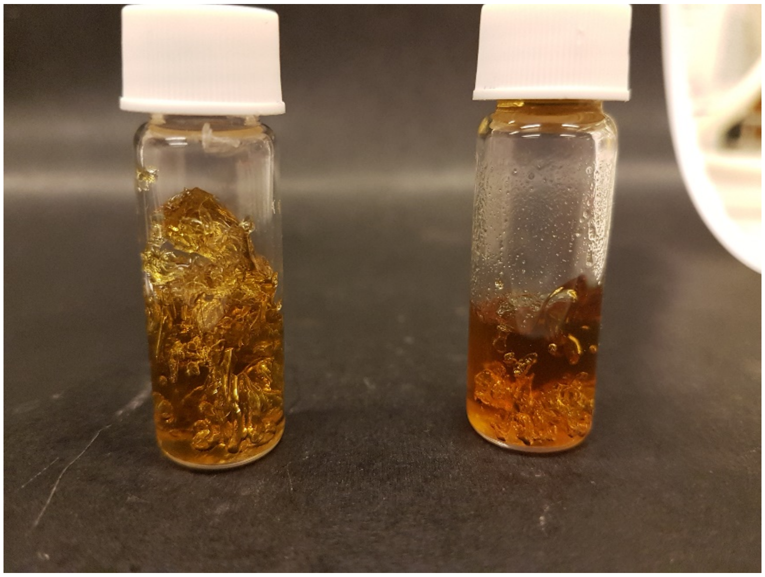

Figure S4. Thermal polymerization gelling at 70°C with C-HPG5, Thiocure ETTMP 1300, TATAO and AIBN. (Left) In methanol and (Right) in 1-propanol.

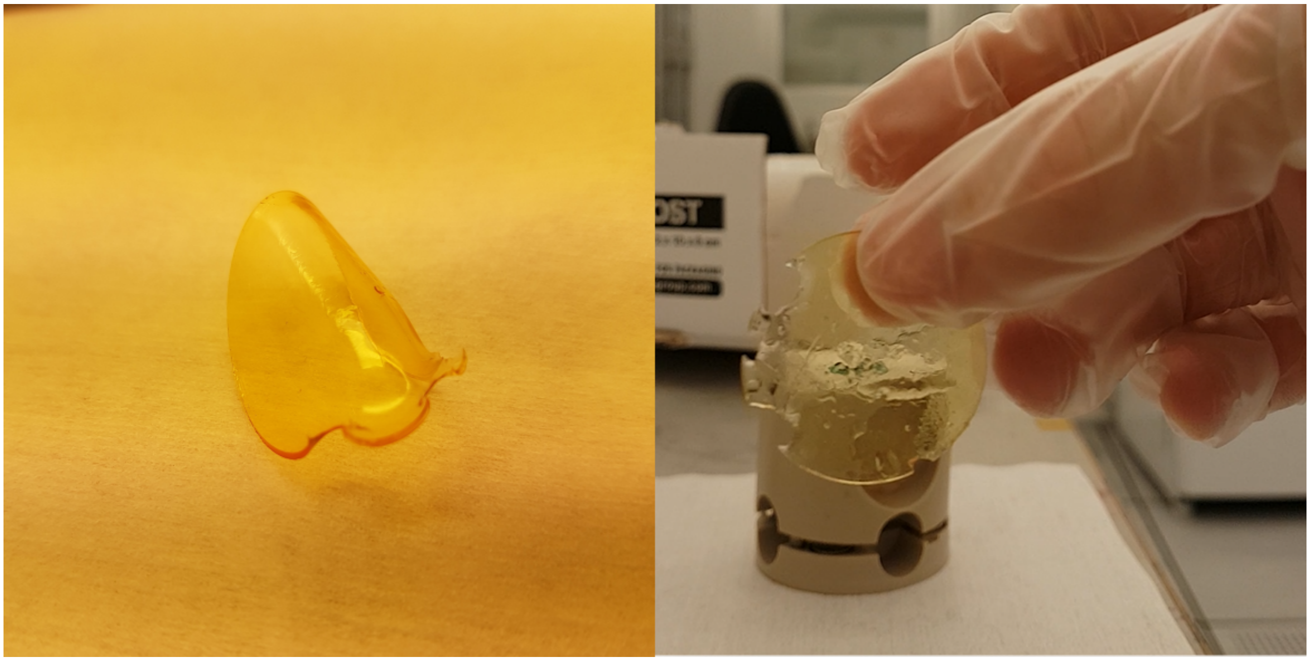

Figure 5. Left: Transparent cross-linked C-HPG5 membrane with flexibility. Right: Water swelling resulting in a more rigid membrane.

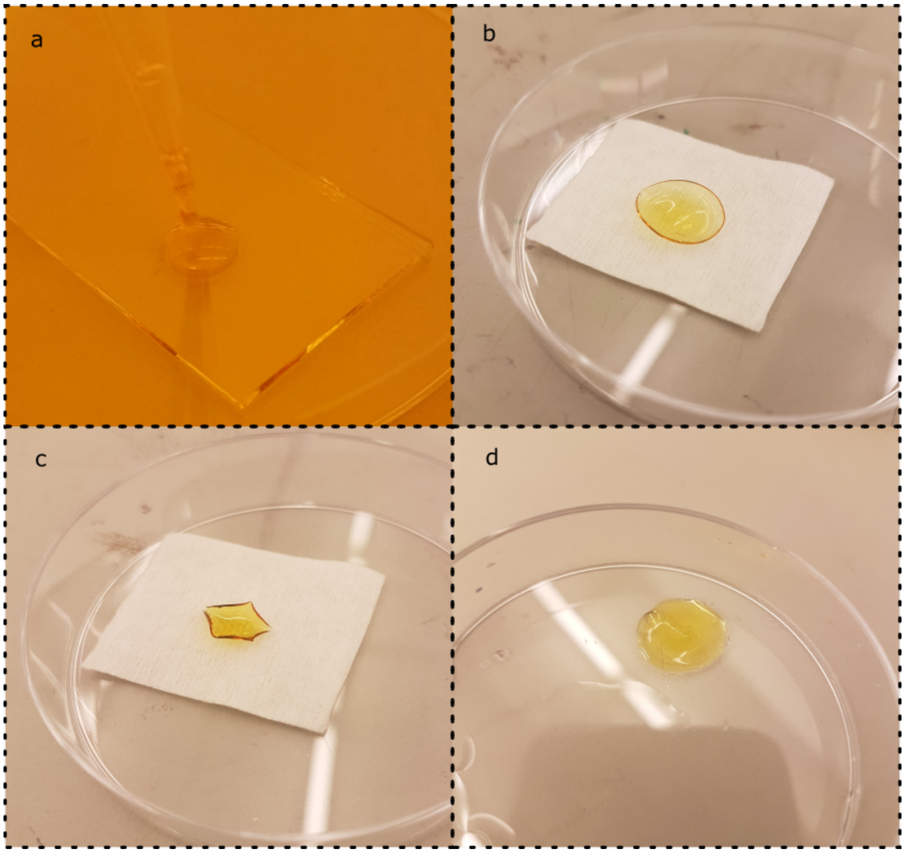

Figure S6. (a) Photo-initiated C-HPG5 methanol/water solution (Table 2. UVC4) drop-casted on glass substrate. (b) Cross-linked hydrated membrane peeled off from the surface (c) and dried in oven. (d) Rehydrated membrane in water.

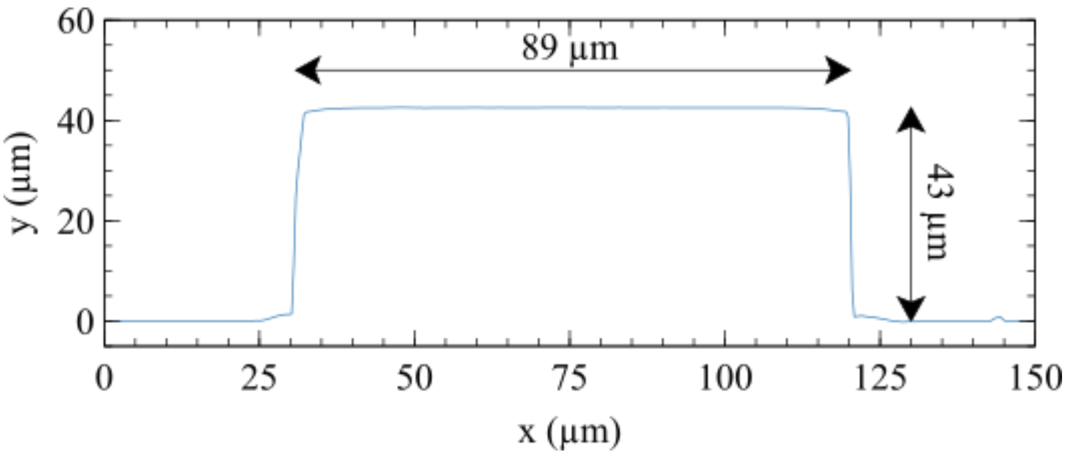

Figure S7. Channel Profile – 8 mm length channel. Optical profilometer (Sensofar PLu Neox) using a 50x confocal objective.

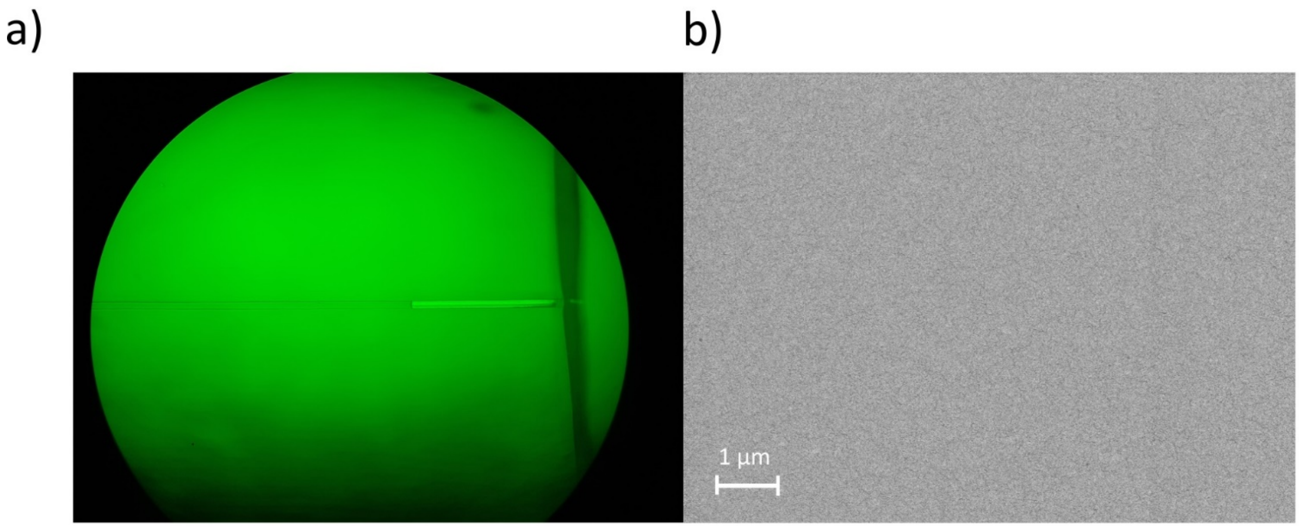

Figure S8. a) Capillary filling process of C-HPG5 monitored by microscopy. b) Scanning electron microscopy image of a fabricated, non-encapsulated C-HPG5 capillary channel.

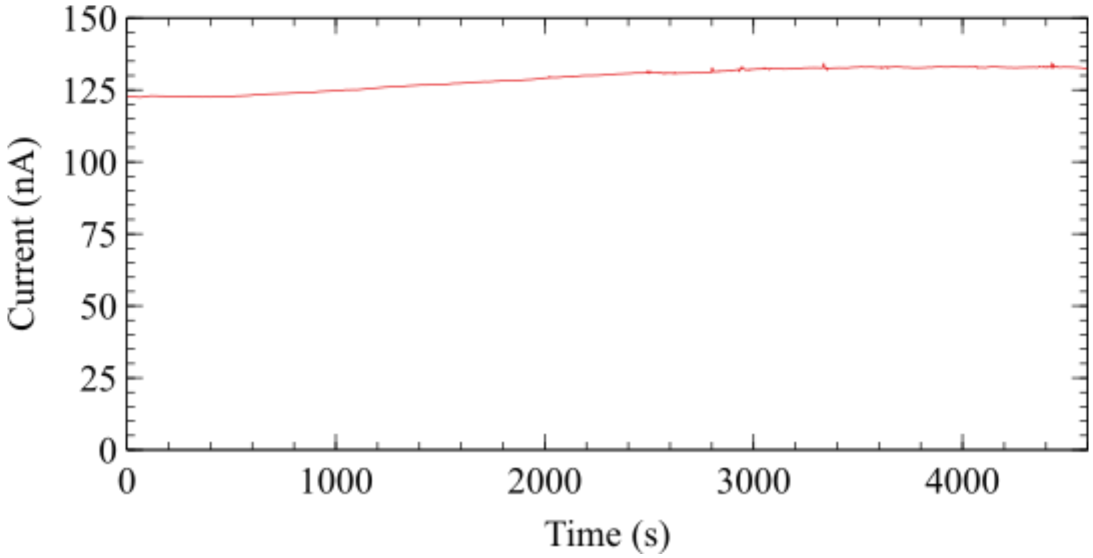

Figure S9. C-HPG5 microchannel in 0.1 M NaCl, applying 1 V.
